# Imidazole-based Potential Bi- and Tridentate Nitrogen Ligands: Synthesis, Characterization and Application in Asymmetric Catalysis

**DOI:** 10.3390/molecules13092326

**Published:** 2008-09-25

**Authors:** Roman Sívek, Filip Bureš, Oldřich Pytela, Jiří Kulhánek

**Affiliations:** Institute of Organic Chemistry and Technology, Faculty of Chemical Technology, University of Pardubice, nám. Čs. legií 565, Pardubice, CZ-532 10, Czech Republic

**Keywords:** Imidazole, Nitrogen ligands, Asymmetric catalysis

## Abstract

Twelve new imidazole-based potential bi- and tridentate ligands were synthesized and characterized. Whereas in the first series the α-amino acid and imidazole moieties were linked by an amino bond, in the second series the tridentate ligands, containing two imidazole groups, were separated by an amide bond. The first series was obtained by the reductive amination of 2-phenylimidazole-4-carboxaldehyde with α‑amino acid esters. The tridentate ligands were prepared from 2‑phenylimidazole-4-carboxylic acid and chiral amines. In the Henry reaction, the amines were revealed as a more reactive species than the less nucleophilic amides, however the enantiomeric excesses were generally poor.

## Introduction

A remarkable effort has been devoted by organic chemists over the past 10 years to the design, synthesis, characterization and applications of diverse chiral imidazole-based derivatives [1,2,3,4,5,6,7,8,9,10,11]. These five-membered heterocyclic compounds are mainly being explored for their interesting physicochemical and biological properties, thermal and chemical robustness, acid-base character and possible tautomerism, and last but not least, for their easy synthesis and possible manifold functionalization. Imidazole is frequently found as part of a large number of biologically and medicinally significant substances [12,13] e.g. histidine and its derivatives or as part of the purine skeleton [14]. More recently, imidazole and its derivatives became of interest due to their ability to bind various transition metals [15,16]. In such complexes, the imidazole with its two nitrogen atoms serves as a coordination part of the molecule whereas the chiral auxiliaries at positions 1, 2, 4 or 5 provide an overall asymmetrical environment. This way, designed complexes were able to perform as promising candidates for application in a wide range of asymmetric reactions involving e.g. the Henry reaction [17], conjugate addition [18], addition of dialkylzinc to aldehydes [19], allylation [20], epoxidation and cyclopropanation [21], oxidation [22] or transfer hydrogenation [23].

**Scheme 1:**
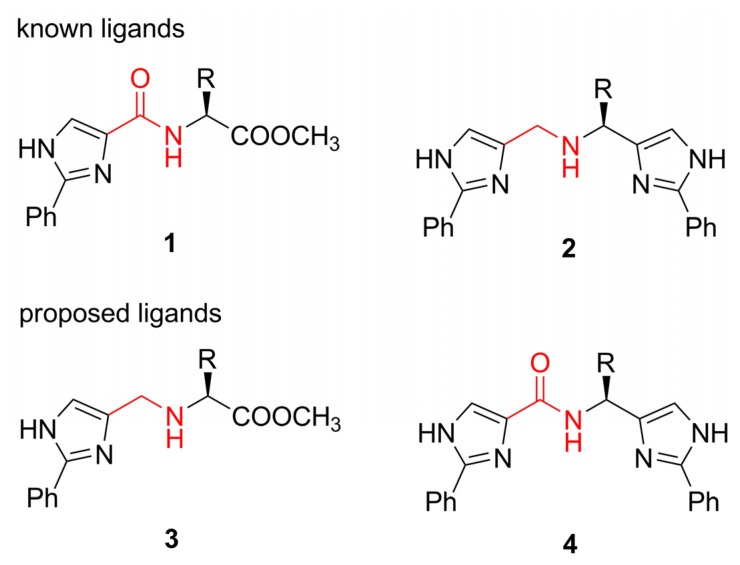
Known and newly proposed imidazole-based ligands.

Several readily available enantiopure precursors such as α-amino acids [2,4,5,7], chiral amines [9], 1,2-amino alcohols [6,10] or α-(acetyloxy)aldehydes [8] were already utilized as a convenient starting material in the synthesis of the chiral imidazole derivatives. Recently, we reported on the synthesis and application of the 2-phenylimidazolecarboxamides **1** featuring an amino acid motive [24], as well as on the tridentate ligands **2** prepared from α-amino acids containing two imidazole groups linked through an amino bond [17] (Scheme 1). Having established the synthesis and catalytic activity of these two classes of compounds bearing either amino or amide bonds and featuring motives from essential α‑amino acids, we turned our attention to the synthesis and investigation of their counterparts. Structures of the two newly proposed ligand series are also depicted in Scheme 1. Whereas the first class of compounds **3** comprises molecules bearing an amino acid residue linked by an amino bond, the second **4** contains two imidazole groups linked by an amide bond. Here we report the synthesis of the two new ligand classes **3** and **4**, thus allowing a systematical investigation of the amino vs. amide linkers between the α-amino acid residues and the chelating imidazole moiety (ie. comparing series **1** vs. **3** and **2** vs. **4**, respectively) and their influence on the catalytic activity in chosen asymmetric reactions.

## Results and Discussion

### Ligand synthesis

**Scheme 2:**
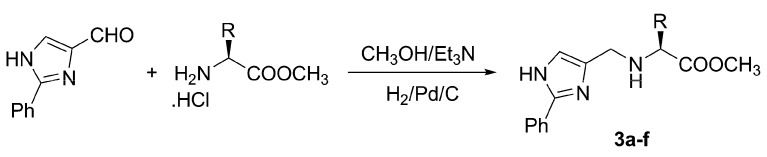
The reductive amination leading to ligands **3a-f**.

Our synthetic approach to the first series **3** resembles those used for the synthesis of the precedent tridentate ligands [17]. This reaction involves a simple condensation between 2-phenylimidazole-4-carboxaldehyde and free amines (α-amino acid esters) affording unstable imines that were directly reduced *in‑situ* using the H_2_/Pd/C system (Scheme 2, Table 1). The starting 2‑phenylimidazole-4-carboxaldehyde is accessible via condensation of dihydroxyacetone with benzamidine in liquid ammonia and oxidation of the resulting hydroxymethyl intermediate with concentrated nitric acid [25]. The amino acid esters hydrochlorides were prepared by a known method [26], whereas the free amino bases were liberated *in-situ* using triethylamine.

**Table 1:** Bidentate ligands **3a-f**.

Comp.	R / Source of chirality	Yield [%]	e.e. [%]	[α]_D_^20^ (*c* 0.05, CH_3_OH)
**3a**	CH_3 _/ (*S*)-Alanine	56	> 95	-8.9
**3b**	CH(CH_3_)_2 _/ (*S*)-Valine	73	> 95	-22.8
**3c**	CH_2_CH(CH_3_)_2 _/ (*S*)-Leucine	34	> 95	-22.0
**3d**	CH(CH_3_)CH_2_CH_3 _/ (*S*)-Isoleucine	38	> 95	-9.2
**3e**	CH_2_Ph / (*S*)-Phenylalanine	66	> 95	-13.4
**3f**	Ph / (*R*)-Phenylglycine	23	> 95	-16.7

**Scheme 3:**
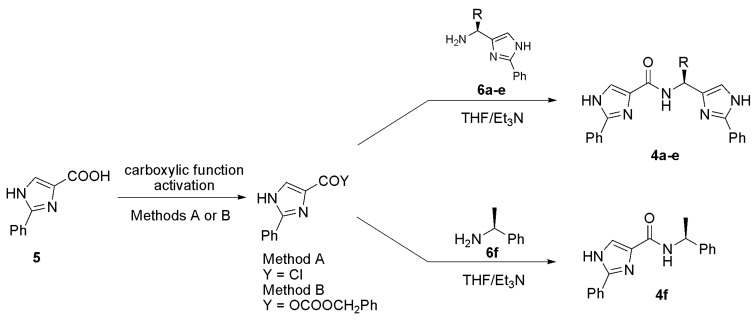
Synthesis of tridentate ligands **4a-e** and ligand **4f**.

Synthesis of the second series **4** started from 2-phenyl-4-carboxylic acid **5** and its activation through acylchlorides (Method A) or mixed anhydrides (Method B, see the Experimental section for more details). Although alternative and more convenient methods for activation of the carboxylic function are well known (e.g. transformation into esters or *in-situ* activation using DCC or CDI), we found these methods unfeasible for **5** [24]. Thus, only **5** activated in the two ways mentioned could be condensed with the chiral amines **6a-e** (Scheme 3, Table 2) obtained from the corresponding *N*‑Cbz-α-amino acids and their transformation into the corresponding α‑diazoketones and α‑bromoketones, respectively, followed by condensation with benzamidine. Finally, Cbz-group removal afforded the desired free amines **6a-e** [4]. In addition, the commercially available (*S*)‑1‑phenylethanamine **6f** was employed as the starting chiral amine as well as affording the bidentate ligand **4f**.

Comparing both methods, Method B utilizing mixed anhydrides was operationally simpler, providing also higher yields, while the yields were solely affected by the undesired formation of the carbamic function on the imidazole nitrogen. Both the activating or condensing steps require careful pH control. Triethylamine as a base maintained the free reactive amino group while scavenging the hydrogen chloride produced during both reactions. The optimal pH value was revealed to be about 9 (possible risk of racemization at higher pH values).

**Table 2:** Tridentate **4a-e** and bidentate ligand **4f**.

Comp.	R / Source of chirality	Yield^[a]^ [%]	e.e. [%]	[α]_D_^20^ (*c* 0.05, CH_3_OH)
**4a**	CH_3 _/ (*S*)-Alanine	23/24	> 95	+95.6
**4b**	CH(CH_3_)_2 _/ (*S*)-Valine	30/35	> 95	+48.0
**4c**	CH_2_CH(CH_3_)_2 _/ (*S*)-Leucine	16/25	> 95	+48.8
**4d**	CH(CH_3_)CH_2_CH_3 _/ (*S*)-Isoleucine	13/34	> 95	+36.0
**4e**	CH_2_Ph / (*S*)-Phenylalanine	17/22	> 95	+33.0
**4f**	CH_3_ / (*S*)-1-Phenylethanamine	44/42	> 95	+142.0

^[a]^ Isolated yields for Methods A/B

### Asymmetric catalysis

**Scheme 4:**
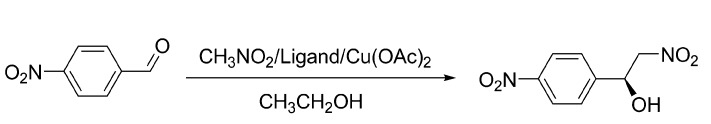
Asymmetric version of the Henry reaction.

Enantioselectivities of the ligands prepared were examined in the Henry reaction [27]. Its asymmetric version involves a reaction between aldehyde and nitroalkane catalyzed by the chiral ligands chelating mainly copper (II) [28,29], zinc [30] or rare earth metal salts [31] (Scheme 4).

This reaction serves as a basic screening of the enantioselectivity giving the first insight into the catalytic behaviour of the studied ligands. The yields and enantiomeric excesses (ee) achieved for ligands **3a-f** and **4a-f** as well as for the precedent ligands **1a-f** [24] and **2a-c, 2e** [17] are summarized in Table 3. When comparing the attained chemical yields for series **1** and **3**, we can deduce that the amines (series **3**) are more efficient catalysts/bases than less nucleophilic amides (series **1**).

**Table 3:** The Henry reaction – yields and enantiomeric excesses.

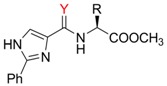					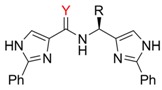				
Lig.	R	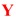	Yield [%]	ee [%]	Lig.	R	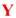	Yield [%]	ee [%]
**3a**	CH_3_	H, H	98	10	**4a**	CH_3_	O	94	10
**3b**	CH(CH_3_)_2_	H, H	96	7	**4b**	CH(CH_3_)_2_	O	91	6
**3c**	CH_2_CH(CH_3_)_2_	H, H	94	15	**4c**	CH_2_CH(CH_3_)_2_	O	94	14
**3d**	CH(CH_3_)CH_2_CH_3_	H, H	97	10	**4d**	CH(CH_3_)CH_2_CH_3_	O	89	8
**3e**	CH_2_Ph	H, H	95	14	**4e**	CH_2_Ph	O	99	15
**3f**	Ph	H, H	93	9	**4f**	see Scheme 3	O	91	5
**1a** ^[a]^	CH_3_	O	79	1	**2a** ^[b]^	CH_3_	H, H	94	13
**1b** ^[a]^	CH(CH_3_)_2_	O	84	3	**2b** ^[b]^	CH(CH_3_)_2_	H, H	95	13
**1c** ^[a]^	CH_2_CH(CH_3_)_2_	O	85	8	**2c** ^[b]^	CH_2_CH(CH_3_)_2_	H, H	96	15
**1d** ^[a]^	CH(CH_3_)CH_2_CH_3_	O	91	4	**2d** ^[c]^	CH(CH_3_)CH_2_CH_3_	H, H	-	-
**1e** ^[a]^	CH_2_Ph	O	90	3	**2e** ^[b]^	CH_2_Ph	H, H	96	19
**1f** ^[a]^	Ph	O	70	4					

^[a]^ Taken from Ref. [24] ^[b]^ Taken from Ref. [17] ^[c]^ No available data.

Although the enantiomeric excesses for both series are poor, the attained ee values have the same trend as those for the chemical yields. As a general trend, the attained ee’s increase throughout the data in Table 3 along with an increased bulk of the substituent R (e.g. the highest ee measured for derivatives with bulky benzyl group – ligands **3e** and **4e**). Comparison of the chemical yields for series **2** and **4** is less straightforward. The catalytic activity/basicity of the tridentate ligands is most likely given by the presence of two imidazole moieties. However, the attained enantiomeric excesses were slightly higher for the amines (series **2**). 

## Conclusions

**Figure 1:**
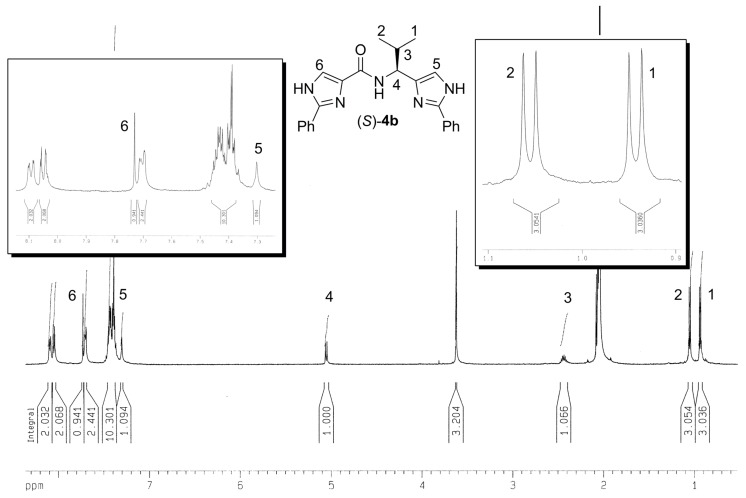
^1^H-NMR spectra of (*S*)**-4b** measured with (*R*)-Mosher’s acid (*d_6_*-acetone) used for the ee’s determination.

**Figure 2:**
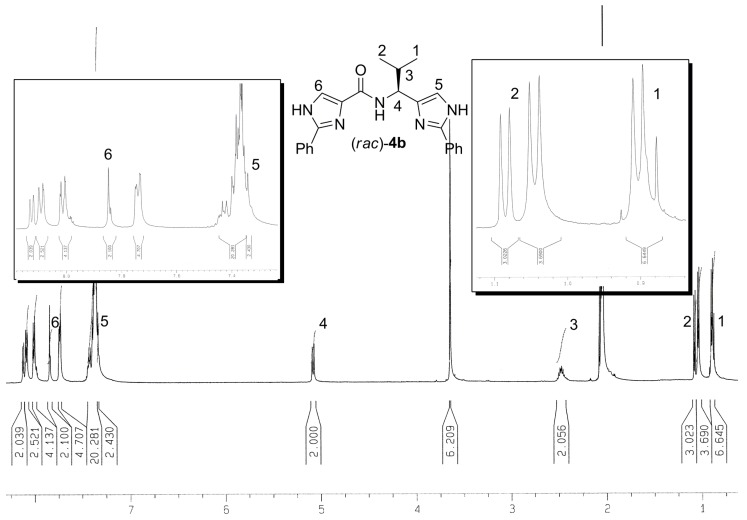
^1^H-NMR spectra of (*rac*)**-4b** measured with (*R*)-Mosher’s acid (*d_6_*-acetone) used for the ee’s determination (compare in particular the 2-H signals with (*S*)-**4b** on the Figure 1).

We have synthesized two new classes of compounds bearing either amino or amide bonds. The first series **3**, where an imidazole ring and α-amino acid ester auxiliaries were linked via an amine, was obtained by the simple reductive amination. The second series **4** was comprised of tridentate ligands containing two 2-phenylimidazole groups bonded through an amide bond. Tridentate ligands **4e-f** were prepared from the corresponding 2-phenyl-4-carboxylic acid employing two activation methods followed by condensation with either synthetically accessible or commercially available amines. The method of activation utilizing benzylchloroformate (Method B) proved to be more efficient than the method proceeding through the corresponding acylchloride (Method A). The optical purities of compounds **3a**‑**f** as well as **4a**-**f** preserve those from the starting α-amino acids or amines used (as determined by ^1^H-NMR spectra measured with Mosher’s acid; for representative ^1^H-NMR spectra see Figure 1 and Figure 2 ).

The enantioselectivity of both ligand series were examined in the Henry reaction. Whereas the amines as well as the amides were able to catalyze the reaction, both compared amine series (**2** and **3**) revealed to be more efficient catalysts (stronger bases), while higher yields were observed. In general, the attained enantiomeric excesses were poor nevertheless higher ee’s were measured for the amines as well as for the ligands bearing bulkier substituents.

## Experimental

### General

The 2-phenylimidazole-4-carbaldehyde [25], α-amino acid esters [26], 2-phenylimidazole-4-carboxylic acid (**5**) [24] and chiral amines **6a-e** [4] were synthesized according to literature procedures. (*R*)‑Mosher’s acid refers to (*R*)-(+)-α-methoxy-α-trifluoromethylphenylacetic acid (Aldrich). The Henry reaction was carried out under the conditions given in [17]. Reagents and solvents (reagent grade) were purchased from Aldrich or Fluka and used as received. THF was freshly distilled from Na/benzophenone under N_2_. Evaporation and concentration *in vacuo* were performed at water aspirator pressure. The reductive aminations were carried out in a ROTH pressure vessel. Column chromatography (CC) was carried out with SiO_2_ 60 (particle size 0.040-0.063 mm, 230-400 mesh; Merck) and commercially available solvents. Thin-layer chromatography (TLC) was conducted on aluminium sheets coated with SiO_2_ 60 F254 obtained from Merck, with visualization by UV lamp (254 or 360 nm). Melting points (M.p.) were measured on a Büchi B-540 melting-point apparatus in open capillaries and are uncorrected. ^1^H- and ^13^C-NMR spectra were recorded in CD_3_OD at 500 MHz or 125 MHz, respectively, with Bruker AVANCE 500 instrument at 20 °C. Chemical shifts are reported in ppm relative to the signal of Me_4_Si. Residual solvent signals in the ^1^H and ^13^C-NMR spectra were used as an internal reference (CD_3_OD – 3.31 and 49.15 ppm for ^1^H- and ^13^C-NMR, respectively). Coupling constants (*J*) are given in Hz. The apparent resonance multiplicity is described as s (singlet), br s (broad singlet), d (doublet), t (triplet), q (quartet) and m (multiplet). 2-Phenyl protons in compounds **3a-f** and **4a-f** were marked as Ar*H*. 5-Imidazole protons in compounds **4a-e** were marked as *H*_ImL_/*H*_ImR_ (left/right imidazole ring according to the scheme in Table 3). Additional NMR techniques such as ^1^H-^1^H COSY, HMBC, and HMQC spectra were further used for regular signal assignment (especially for distinguishing *H*_ImL_ and *H*_ImR_ signals in compounds **4a**-**f**, and for regular carbon assignment). Optical rotation values were measured on a Perkin Elmer 341 instrument, concentration *c* is given in g/100 mL CH_3_OH. The enantiomeric excesses were determined by chiral HPLC analysis on a Daicel Chiracel OB column and simultaneously deduced from [α] values [17].

### General method for reductive amination. Preparation of **3a-f**

Catalyst - Pd/active carbon (0.05 g; 10%, Aldrich^®^) was added to a solution of 2-phenylimidazole-4-carbaldehyde (0.40 g; 2.3 mmol) and α‑amino acid ester (2.3 mmol) in dry methanol (15 mL) and triethylamine (0.35 mL; 2.4 mmol). The solution was degassed and saturated with hydrogen in an autoclave at 1 MPa at 55 °C for 2 h. The catalyst was filtered off, washed with methanol and the filtrate concentrated *in vacuo*. The crude product was purified by CC (SiO_2_; ethyl acetate/methanol 4:0.7).

*(2S)-Methyl 2-[(2-phenyl-1H-imidazol-4-yl)methylamino]propanoate* (**3a**). Prepared from (*S*)-alanine methyl ester hydrochloride in 56% yield; m.p. 145-146 ºC; [α]_D_^20^ = -8.9 (*c* 0.05, CH_3_OH); ^1^H-NMR: *δ* = 1.34 (3H, d, *J* = 7.0, C*H*_3_), 3.50 (1H, q, *J* = 7.0, C*H*NH), 3.70 (3H, s, OC*H*_3_), 3.77 (1H, d, *J* = 13.8, C*H*_2_NH), 3.83 (H, d, *J* = 13.8, C*H*_2_NH), 7.07 (1H, s, *H*_Im_), 7.37 (1H, t, *J* = 7.4, Ar*H*), 7.44 (2H, t, *J* = 7.4, Ar*H*,), 7.85 (2H, d, *J* = 7.4, Ar*H*); ^13^C-NMR: *δ* = 18.5 (*C*H_3_), 44.5 (*C*H_2_NH), 52.7 (O*C*H_3_), 56.60 (*C*HNH), 122.1 (*C*5_Im_), 126.5 (ArH), 130.0 (ArH), 130.1 (ArH), 131.4 (Ar_q_), 137.6 (*C*4_Im_), 148.3 (*C*2_Im_), 176.4 (*C*OOCH_3_); Elemental analysis (%) calcd. for C_14_H_17_N_3_O_2_: C 64.85, H 6.61, N 16.20; found: C 64.90, H 6.58, N 16.23.

*(2S)-Methyl 3-methyl-2-[(2-phenyl-1H-imidazol-4-yl)methylamino]butanoate* (**3b**). Prepared from (*S*)-valine methyl ester hydrochloride in 73% yield; m.p. 141-142 ºC; [α]_D_^20^ = -22.8 (*c* 0.05, CH_3_OH); ^1^H-NMR: *δ* = 0.91 (3H, d, *J* = 6.9, (C*H*_3_)_2_), 0.94 (3H, d, *J* = 6.9, (CH_3_)_2_), 1.91-1.97 (1H, m, C*H*(CH_3_)_2_), 3.11 (1H, d, *J* = 5.8, C*H*NH), 3.65 (3H, s, OCH_3_), 3.67 (1H, d, *J* = 14.0, C*H*_2_NH), 3.77 (1H, d, *J* = 14.0, C*H*_2_NH), 6.99 (1H, s, *H*_Im_), 7.36 (1H, t, *J* = 7.5, Ar*H*), 7.42 (2H, t, *J* = 7.5, Ar*H*), 7.84 (2H, d, *J* = 7.5, Ar*H*); ^13^C-NMR: *δ* 19.3 ((*C*H_3_)_2_), 32.7 (*C*H(CH_3_)_2_), 45.3 (*C*H_2_NH), 52.4 (O*C*H_3_), 67.5 (*C*HNH), 122.2 (*C*5_Im_), 126.4 (ArH), 129.8 (ArH), 130.1 (ArH), 131.6 (Ar_q_), 138.2 (*C*4_Im_), 148.1 (*C*2_Im_), 176.5 (*C*OOCH_3_); Elemental analysis (%) calcd. for C_16_H_21_N_3_O_2_: C 66.88, H 7.37, N 14.62; found: C 66.91, H 7.33, N 14.60.

*(2S)-Methyl 4-methyl-2-[(2-phenyl-1H-imidazol-4-yl)methylamino]pentanoate* (**3c**). Prepared in 34% yield from (*S*)-leucine methyl ester hydrochloride; m.p. 115-117 °C; [α]_D_^20^ = -22.0 (*c* 0.05, CH_3_OH); ^1^H-NMR: *δ =* 0.85 (3H, d, *J* = 6.6, (C*H*_3_)_2_), 0.92 (3H, d, *J* = 6.6, (C*H*_3_)_2_), 1.47-1.52 (2H, m, C*H_2_*CH), 1.66-1.70 (1H, m, C*H*(CH_3_)_2_), 3.38 (1H, t, *J* = 7.2, C*H*NH), 3.67 (3H, s, OC*H*_3_), 3.68 (1H, d, *J* = 13.9, C*H_2_*NH), 3.79 (1H, d, *J* = 13.9, C*H_2_*NH), 7.01 (1H, s, *H*_Im_), 7.37 (1H, t, *J* = 7.5, Ar*H*), 7.44 (2H, t, *J* = 7.5, Ar*H*), 7.84 (2H, d, *J* = 7.2, Ar*H*); ^13^C-NMR: *δ* = 23.0 (*C*H_3_)_2_), 23.1 (*C*H_3_)_2_), 26.2 (*C*H(CH_3_)_2_), 43.7 (*C*H*_2_*CH), 45.0 (br, *C*H_2_NH), 52.4 (O*C*H_3_), 60.2 (*C*HNH), 122.2 (*C*5_Im_), 126.5 (ArH), 129.9 (ArH), 130.1 (ArH), 131.6 (Ar_q_), 148.2 (*C*2_Im_), 177.2 (*C*OOCH_3_), *C*4_Im_ is missing; Elemental analysis (%) calcd. for C_17_H_23_N_3_O_2_: C 67.75, H 7.69, N 13.94; found: C 67.73, H 7.72, N 13.98.

*(2S,3S)-Methyl* *3-methyl-2-[(2-phenyl-1H-imidazol-4-yl)methylamino]pentanoate* (**3d**). Prepared from (2*S*,3*S*)-isoleucine methyl ester hydrochloride in 38% yield; m.p. 93-98 °C; [α]_D_^20^ = -9.2 (*c* 0.05, CH_3_OH); ^1^H-NMR: *δ* = 0.87-0.93 (6H, m, CHC*H_3_* and CH_2_C*H_3_*), 1.17-1.24 (1H, m, C*H_2_*CH_3_), 1.50-1.55 (1H, m, C*H_2_*CH_3_), 1.70-1.72 (1H, m, C*H*CH_3_), 3.23 (1H, d, *J* = 5.7, C*H*NH), 3.65 (3H, s, OC*H*_3_), 3.67 (1H, d, *J* = 14.0, C*H_2_*NH), 3.77 (1H, d, *J* = 14.0, C*H_2_*NH), 6.99 (1H, s, *H*_Im_), 7.36 (1H, t, *J* = 7.4, Ar*H*), 7.43 (2H, t, *J* = 7.4, Ar*H*), 7.84 (2H, d, *J* = 7.8, Ar*H*); ^13^C-NMR: *δ* = 12.0 (CH_2_*C*H_3_), 15.9 (CH*C*H_3_), 27.1 (*C*H_2_CH_3_), 39.7 (*C*HCH_3_), 45.3 (*C*H_2_NH), 52.1 (O*C*H_3_), 66.1 (*C*HNH), 122.2 (*C*5_Im_), 126.7 (ArH), 129.9 (ArH), 130.1 (ArH), 131.6 (Ar_q_), 148.2 (*C*2_Im_), 176.4 (*C*OOCH_3_), *C*4_Im_ is missing; Elemental analysis (%) calcd. for C_17_H_23_N_3_O_2_: C 67.75, H 7.69, N 13.94; found: C 67.72, H 7.73, N 13.96.

*(2S)-Methyl 3-phenyl-2-[(2-phenyl-1H-imidazol-4-yl)methylamino]propanoate* (**3e**). Prepared from (*S*)-phenylalanine methyl ester hydrochloride in 66% yield; m.p. 165-166 °C; [α]_D_^20^ = -13.4 (*c* 0.05, CH_3_OH); ^1^H-NMR: *δ* = 2.94 (2H, 2, *J* = 9.7, C*H*_2_Ph), 3.58 (3H, s, OC*H_3_*), 3.61 (1H, t, *J* = 7.1, C*H*NH), 3.68 (1H, d, *J* = 14.0, C*H*_2_NH), 3.78 (1H, d, *J* = 14.0, C*H_2_*NH), 6.92 (1H, s, *H*_Im_), 7.14-7.30 (5H, m, Ph), 7.36 (1H, t, *J* = 7.0, Ar*H*), 7.43 (2H, t, *J* = 7.7, Ar*H*), 7.81 (2H, d, *J* = 7.3, Ar*H*); ^13^C-NMR: *δ* = 40.4 (*C*H_2_Ph), 45.2 (br, *C*H_2_NH), 52.3 (O*C*H_3_), 63.3 (*C*HNH), 122.3 (*C*5_Im_), 126.5 (ArH), 127.9 (Ph), 129.6 (Ph), 129.9 (ArH), 130.1 (ArH), 130.4 (Ph), 131.5 (Ar_q_), 138.6 (Ph_q_), 148.2 (*C*2_Im_), 176.0 (*C*OOCH_3_), *C*4_Im_ is missing; Elemental analysis (%) calcd. for C_20_H_21_N_3_O_2_: C 71.62, H 6.31, N 12.53; found: C 71.65, H 6.25, N 12.59.

*(2S)-Methyl 2-phenyl-2-[(2-phenyl-1H-imidazol-4-yl)methylamino]ethanoate* (**3f**). Synthesized from (*S*)-glycine methyl ester hydrochloride in 23% yield; m.p. 157-158 °C; [α]D^20^ = -16.7 (*c* 0.05, CH_3_OH); ^1^H-NMR: *δ* = 3.63 (3H, s, OC*H*_3_), 3.73 (2H, s, C*H*2NH), 4.47 (1H, s, C*H*NH), 7.00 (1H, s, *H*_Im_), 7.28-7.44 (8H, m, Ar*H* and Ph), 7.84 (2H, d, *J* = 7.4, Ar*H*); ^13^C-NMR: *δ* = 44.3 (*C*H_2_NH), 52.8 (O*C*H_3_), 65.7 (*C*HNH), 122.4 (*C*5_Im_), 126.5 (ArH), 129.7 (Ph), 129.5 (Ph), 129.9 (Ph), 130.0 (ArH), 130.1 (ArH), 131.5 (Ar_q_), 139.1 (Ph_q_), 148.3 (*C*2_Im_), 174.6 (*C*OOCH3), *C*4_Im_ is missing; Elemental analysis (%) calcd. for C_19_H_19_N_3_O_2_: C 71.01, H 5.96, N 13.08. Found: C 71.07, H 6.03, N 12.99.

### General procedure for the preparation of **4a-f**

#### *Method A* 

Thionyl chloride (5 mL; 69 mmol) was added dropwise to a stirred and ice-cooled suspension of **5** (1.0 g; 5.3 mmol) in dry THF (200 mL). The reaction mixture was refluxed for 6 h, all of the volatiles evaporated *in vacuo* and the crude acylchloride used in the next step without further purification. A solution of the amine **6a-f** (4.7 mmol) in dry THF (30 mL) was added dropwise to a stirred and ice-cooled solution of the above acylchloride (1 g; 4.8 mmol) in dry THF (180 mL), followed by gradual addition of triethylamine (1.5 mL, 10.7 mmol) as rapidly as pH doesn’t exceed 7. The reaction mixture was stirred for 12 h at 25 ºC, the precipitated triethylamine hydrochloride filtered off, the filtrate concentrated *in vacuo* and the residue purified by CC (SiO_2_; ethyl acetate/methanol 4:0.7).

#### *Method B* 

Benzylchlorofomate (0.97 mL 6.8 mmol) was added dropwise to a solution of **5** (1.0 g; 5.3 mmol) and triethylamine (1.5 mL; 10.8 mmol) in dry THF (200 mL) under N_2_ at -10º. The reaction mixture was stirred for an additional 30 min whereupon a solution of amine **6a-f** (5.2 mole) in dry THF (30 mL) was added. The reaction was stirred for 12 h at 25 ºC, the precipitated triethylamine hydrochloride filtered off, the filtrate concentrated *in vacuo* and the crude product purified by CC (SiO_2_; ethyl acetate/methanol 4:0.7).

*(1S)-2-Phenyl-N-[1-(2-phenyl-1H-imidazol-4-yl)ethyl]-1H-imidazole-4-carboxamide* (**4a**). This compound was synthesized from amine **6a** in yields of 23 (method A) and 24% (method B), respectively; m.p. 134-135 °C; [α]_D_^20^ = +95.6 (*c* 0.05, CH_3_OH). ^1^H-NMR: *δ* = 1.62 (3H, d, *J* = 6.9, C*H*_3_), 5.32 (1H, q, *J* = 6.9, C*H*NH), 7.08 (1H, s, *H*_ImR_), 7.31-7.43 (6H, m, Ar*H*), 7.73 (1H, s, *H*_ImL_), 7.84 (2H, d, *J* = 7.3, Ar*H*), 7.89 (2H, d, *J* = 7.1, Ar*H*). ^13^C-NMR: *δ* = 21.2 (*C*H_3_), 44.1 (*C*HNH), 118.4 (*C*5_ImR_), 123.4 (*C*5_ImL_), 126.7 (ArH), 126.9 (ArH), 129.9 (ArH), 130.0 (ArH), 130.1 (ArH), 130.5 (ArH), 131.0 (Ar_q_), 131.4 (Ar_q_), 137.2 (*C*4_ImL_), 143.1 (*C*4_ImR_), 148.4 (*C*2_ImR_), 148.9 (*C*2_ImL_), 164.2 (*C*ONH). Elemental analysis (%) calcd. for C_21_H_19_N_5_O: C 70.57, H, 5.36; N, 19.59. Found: C, 70.55; H, 5.40; N, 19.54.

*(1S)-N-[2-Methyl-1-(2-phenyl-1H-imidazol-4-yl)propyl]-2-phenyl-1H-imidazole-4-carboxamide* (**4b**). This compound was synthesized from amine **6b** in yields of 30 (method A) and 35% (method B), respectively; m.p. 127-128 °C; [α]_D_^20^ = +48.0 (*c* 0.05, CH_3_OH). ^1^H-NMR: *δ* = 0.95 (3H, d, *J* = 6.7, (C*H*_3_)_2_), 1.06 (3H, d, *J* = 6.7, (C*H*_3_)_2_), 2.29-2.35 (1H, m, C*H*(CH_3_)_2_), 5.04 (1H, d, *J* = 5.8, C*H*NH), 7.09 (1H, s, *H*_ImR_), 7.30-7.44 (6H, m, Ar*H*), 7.74 (1H, s, *H*_ImL_), 7.85 (2H, d, *J* = 7.3, Ar*H*), 7.91 (2H, d, *J* = 7.2, Ar*H*). ^13^C-NMR: *δ* = 19.4 ((*C*H_3_)_2_), 20.4 ((*C*H_3_)_2_), 34.2 (*C*H(CH_3_)_2_), 54.1 (*C*HNH), 119.2 (*C*5_ImR_), 123.1 (*C*5_ImL_), 126.4 (ArH), 126.9 (ArH), 129.9 (ArH), 130.0 (ArH), 130.1 (ArH), 130.5 (ArH), 131.1 (Ar_q_), 131.5 (Ar_q_), 137.5 (*C*4_ImL_), 141.1 (*C*4_ImR_), 148.2 (*C*2_ImR_), 148.8 (*C*2_ImL_), 164.6 (*C*ONH). Elemental analysis (%) calcd. for C_23_H_23_N_5_O: C 71.67, H 6.01, N 18.17. Found: C 71.66, H 5.97, N 18.20.

*(1S)-N-[3-Methyl-1-(2-phenyl-1H-imidazol-4-yl)butyl]-2-phenyl-1H-imidazole-4-carboxamide* (**4c**). This compound was synthesized from amine **6c** in yields of 16 (method A) and 25% (method B), respectively; m.p. 155-157 °C; [α]_D_^20^ = +48.8 (*c* 0.05, CH_3_OH). ^1^H-NMR: *δ* = 0.99 (6H, deceptively t, *J* = 6.2, (C*H*_3_)_2_), 1.66-1.71 (1H, m, C*H*(CH_3_)_2_), 1.87 (2H, t, *J* = 6.9, C*H_2_*CH), 5.35 (1H, t, *J* = 7.5, C*H*NH), 7.07 (1H, s, *H*_ImR_), 7.31-7.45 (6H, m, Ar*H*), 7.73 (1H, s, *H*_ImL_), 7.85 (2H, d, *J* = 7.2, Ar*H*), 7.90 (2H, s, *J* = 7.2, Ar*H*). ^13^C-NMR: *δ* = 22.8 ((*C*H_3_)_2_), 23.3 ((*C*H_3_)_2_), 26.4 (*C*H(CH_3_)_2_), 45.4 (*C*H_2_CH), 46.4 (*C*HNH), 118.7 (*C*5_ImR_), 123.0 (*C*5_ImL_), 126.6 (ArH), 126.9 (ArH), 129.9 (ArH), 130.0 (ArH), 130.1 (ArH), 130.5 (ArH), 131.1 (Ar_q_), 131.6 (Ar_q_), 138.1 (*C*4_ImL_), 142.3 (*C*4_ImR_), 148.4 (*C*2_ImR_), 148.8 (*C*2_ImL_), 165.1 (*C*ONH). Elemental analysis (%) calcd. for C_24_H_25_N_5_O: C 72.16, H 6.31, N 17.53. Found: C 72.15, H 6.36, N 17.50.

*(1S,2S)-N-[2-Methyl-1-(2-phenyl-1H-imidazol-4-yl)butyl]-2-phenyl-1H-imidazole-4-carboxamide* (**4d**). This compound was synthesized from amine **6d** in yields of 13 (method A) and 34% (method B), respectively; m.p. 144-145 °C; [α]_D_^20^ = +36.0 (*c* 0.05, CH_3_OH). ^1^H-NMR: *δ* = 0.93 (3H, d, *J* = 6.7, CHC*H_3_*), 0.96 (3H, t, *J* = 7.5, CH_2_C*H_3_*), 1.25-1.30 (1H, m, C*H_2_*CH_3_), 1.68-1.73 (1H, m, C*H_2_*CH_3_), 2.09-2.14 (1H, m, C*H*CH_3_), 5.09 (1H, d, *J* = 8.2, C*H*NH), 7.09 (1H, s, *H*_ImR_), 7.30-7.46 (6H, m, Ar*H*), 7.73 (1H, s, *H*_ImL_), 7.85 (2H, d, *J* = 7.4, Ar*H*), 7.92 (2H, d, *J* = 7.3, Ar*H*). ^13^C-NMR: *δ* = 11.8 (*C*H_3_CH), 16.6 (*C*H_3_CH_2_), 26.6 (*C*H_2_CH_3_), 40.4 (*C*HCH_3_), 52.8 (*C*HNH), 119.0 (*C*5_ImR_), 123.6 (*C*5_ImL_), 126.6 (ArH), 126.9 (ArH), 129.9 (ArH), 130.0 (ArH), 130.1 (ArH), 130.5 (ArH), 131.1 (Ar_q_), 131.5 (Ar_q_), 137.8 (*C*4_ImL_), 141.3 (*C*4_ImR_), 148.2 (*C*2_ImR_), 148.8 (*C*2_ImL_), 165.0 (*C*ONH). Elemental analysis (%) calcd. for C_24_H_25_N_5_O: C 72.16, H 6.31, N 17.53. Found: C 72.23, H 6.25, N 17.61.

*(1S)-2-Phenyl-N-[2-phenyl-1-(2-phenyl-1H-imidazol-4-yl)ethyl]-1H-imidazole-4-carboxamide* (**4e**). This compound was synthesized from amine **6e** in yields of 17 (method A) and 22% (method B), respectively; m.p. 187-188 °C; [α]_D_^20^ = +33.0 (*c* 0.05, CH_3_OH). ^1^H-NMR: *δ* = 3.23-3.35 (2H, m, C*H*_2_Ph), 5.49 (1H, t, *J* = 7.3, C*H*NH), 6.97 (1H, s, *H*_ImR_), 7.11 (1H, t, *J* = 7.3, Ph), 7.19 (2H, t, *J* = 7.3, Ph), 7.22 (2H, d, *J* = 7.2, Ph), 7.32-7.44 (6H, m, Ar*H*), 7.69 (1H, s, *H*_ImL_), 7.86 (2H, d, *J* = 7.4, Ar*H*), 7.89 (2H, d, *J* = 7.2, Ar*H*). ^13^C-NMR: *δ* = 42.5 (*C*H_2_Ph), 50.1 (*C*HNH), 118.6 (*C*5_ImR_), 123.7 (*C*5_ImL_), 126.6 (ArH), 126.9 (ArH),, 127.6 (Ph), 129.4 (ArH), 129.9 (Ph), 130.0 (ArH), 130.1 (ArH), 130.5 (Ph), 131.1 (Ar_q_), 131.5 (Ar_q_), 137.9 (*C*4_ImL_), 139.5 (Ph_q_), 141.6 (*C*4_ImR_), 148.4 (*C*2_ImR_), 148.8 (*C*2_ImL_), 164.5 (*C*ONH). Elemental analysis (%) calcd. for C_27_H_23_N_5_O: C 74.81, H 5.35, N 16.16. Found: C 74.78, H 5.41, N 16.14.

*(1S)-2-Phenyl-N-(1-phenylethyl)-1H-imidazole-4-carboxamide* (**4f**). This compound was synthesized from commercially available (*S*)-1-phenylethanamine (**6f**) in yields of 44 (method A) and 42% (method B), respectively; m.p. 163-164 °C; [α]_D_^20^ = +142.0 (*c* 0.05, CH_3_OH). ^1^H-NMR: *δ* = 1.55 (3H, d, *J* = 7.0, C*H*_3_), 5.21 (1H, q, *J* = 7.0, C*H*NH), 7.22 (1H, t, *J* = 7.4, Ph), 7.31 (2H, t, *J* = 7.4, Ph), 7.39-7.48 (5H, m, Ar*H* and Ph), 7.72 (1H, s, *H*_Im_), 7.91 (2H, d, *J* = 7.1, Ar*H*). ^13^C-NMR: *δ* = 22.7 (*C*H_3_), 50.2 (*C*HNH), 122.8 (*C*5_Im_), 126.9 (ArH), 127.3 (ArH), 128.3 (Ph), 129.7 (Ph), 130.2 (ArH), 130.6 (Ph), 131.1 (Ar_q_), 145.2 (Ph_q_), 148.6 (*C*2_Im_), 164.2 (*C*ONH). Elemental analysis (%) calcd. for C_18_H_17_N_3_O: C 74.20, H 5.88, N 14.42. Found: C 74.17, H 5.85, N 14.46.
